# Patient-Centered Discharge Criteria and Costs of Total Knee Arthroplasty: A Japanese Study

**DOI:** 10.7759/cureus.82572

**Published:** 2025-04-19

**Authors:** Yoshinori Ishii, Hideo Noguchi, Junko Sato, Ikuko Takahashi, Hana Ishii, Ryo Ishii, Kai Ishii, Shin-ichi Toyabe

**Affiliations:** 1 Orthopaedic Surgery, Ishii Orthopaedic and Rehabilitation Clinic, Gyoda, JPN; 2 Orthopaedics, Ishii Orthopaedic and Rehabilitation Clinic, Gyoda, JPN; 3 Plastic and Reconstructive Surgery, Ishii Orthopaedic and Rehabilitation Clinic, Gyoda, JPN; 4 Orthopaedic Surgery, Shinshu University Hospital, Matsumoto, JPN; 5 Information Science and Biostatistics, Niigata University Hospital, Niigata, JPN

**Keywords:** cost structure, health care costs, japan, length of stay, (los), total knee arthroplasty (tka)

## Abstract

Background

The increasing prevalence of total knee arthroplasty (TKA) owing to population aging imposes a considerable financial burden on healthcare systems. In this study, we analyzed the cost structure of TKA in Japan, where comprehensive cost data beyond hospital length of stay (LOS) are limited.

Methods

We retrospectively analyzed 100 consecutive TKAs (89 patients) for primary osteoarthritis performed by a single surgeon between April 2017 and December 2024. LOS was determined based on patient satisfaction and confidence in daily life, without premature discharge owing to medical reasons. The primary outcome measures were total hospital cost (THC) and its components: admission management (AM), surgery, surgery-related expenses, and rehabilitation. We also evaluated the impact of age, sex, body mass index, operation time, American Society of Anesthesiologists grade, clinical scores, and LOS on these costs.

Results

The median LOS was 31 days, with a median THC of JPY 2,268,700 (approximately USD 15,125, based on an exchange rate of USD 1 to JPY 150). The four components of THC accounted for nearly equal proportions between 24.0 % (585,194/2,268,670) and 25.7 % (582,970/2,268,670). THC was strongly correlated with LOS (r=0.751, p<0.001) and weakly correlated with age (r=0.364, p<0.001). After adjusting for multiple comparisons, LOS, AM, and rehabilitation remained significantly and positively associated with THC.

Conclusions

We observed that in the Japanese fee-for-service healthcare system, where surgical fees are typically fixed, prolonged LOS greatly increased hospital administrative and rehabilitation expenses. Conversely, under patient-centered discharge criteria, the near-equivalent cost structure also suggested the requirement for a certain period of comprehensive inpatient care. These findings underscore the need for novel strategies to optimize LOS and control THC.

## Introduction

With the aging of the Japanese population, the number of total knee arthroplasty (TKA) procedures for osteoarthritis has been increasing, reaching 80,000 cases annually [1]. Whereas the primary goal of TKA is pain relief, the procedure is reported to have additional benefits, such as improved body balance [2], bone mineral density and quality [3,4], reduced arteriosclerosis [5], and increased life expectancy [6] owing to increased physical activity following pain reduction. However, the high cost of TKA imposes a considerable financial burden on the Japanese health care system.

Japan's health care system is a universal health insurance system based on a fee-for-service model. The government sets public prices for medical services and surgical supplies related to TKA, including hospitalization costs, surgical fees, implant costs, and rehabilitation fees, through a national health insurance reimbursement schedule. The increase in medical expenses has made it difficult to maintain this system solely through insurance premiums. Currently, government bonds and other financial support are used to sustain the system owing to the increasing gap between insurance premiums and medical expenses [7]. Therefore, the Japanese government has introduced a lump-sum payment system called the Diagnosis Procedure Combination/Per-Diem Payment System (DPC/PDPS) to control excessive medical care utilization and promote the optimization of medical expenses. As of June 1, 2024, this system is being implemented in 1,786 hospitals [8]. However, there is limited research on the comprehensive medical expenses associated with TKA. Most studies focus on the length of stay (LOS) [9-11]; although LOS is a component of cost, it does not capture the full extent of expenses, such as implant costs, rehabilitation, and in-hospital management.

The purpose of this study was to analyze the cost structure of TKA under a fee-for-service system in which LOS is patient-driven. We aimed to categorize hospital expenses into the following: in-hospital management fees, surgical procedure fees, surgery-related expenses, and in-hospital rehabilitation fees. Our goal was to identify those factors that influence total hospital costs (THC). The clinical importance of this study is that our findings can inform cost-containment strategies and promote efficient resource allocation in medical settings under the current system by analyzing the cost structure of TKA in detail.

## Materials and methods

This study was conducted at the Healthcare Corporation Ashinokai and approved by the Institutional Review Board (ID number: 2024-11), and informed consent was obtained from all patients. This study included 100 knee joints from 89 patients (15 joints in 14 males and 85 joints in 75 females) who underwent primary TKA between April 2017 and December 2024. To ensure a homogeneous study population and minimize confounding factors, we specifically included patients with American Society of Anesthesiologists (ASA) physical status I (21 knees) and II (79 knees) [12]. Patients with severe comorbidities or those classified as ASA III or IV [12] were excluded. All patients underwent surgery for primary osteoarthritis of the knee using the New Jersey low-contact stress total knee system (DePuy Synthes, Warsaw, IN, USA). In principle, patients who desired bilateral knee replacement underwent scheduled staged procedures. Patients who underwent revision TKA or conversion from high tibial osteotomy were not included in this study.

Surgical technique and rehabilitation protocol

All TKA surgeries were performed by a single experienced surgeon (YI) using a standardized technique with a standard medial parapatellar approach. Specifically, under general anesthesia, soft tissue release was performed as needed to obtain the appropriate gap balance based on the mechanical alignment method. To prevent excessive intraoperative bleeding, the MT-720 tourniquet system (Mizuho Medical, Tokyo, Japan) was used, which adjusts pressure in synchronization with systolic blood pressure [13]. The femoral component was fixed without bone cement, and the tibial component was fixed with bone cement. Patellar resurfacing or lateral release was not performed in any case. Antibiotics and analgesics were administered intraoperatively and postoperatively, but thromboprophylaxis was not routinely performed. Regarding pain management, multimodal analgesia, such as peripheral nerve blocks or periarticular infiltration analgesia, was not applied. These techniques may have limitations such as an increased risk of falls, delayed rehabilitation after nerve blocks, and variability in outcomes owing to infiltration techniques in periarticular infiltration analgesia.

Postoperative rehabilitation was started after the first dressing change on the first postoperative day. Under the supervision of a physical therapist, weight-bearing with crutches was allowed as tolerated by the patient. Patients received at least two hours of physical therapy daily, comprising isometric exercises, passive range of motion (ROM), active-assisted ROM, muscle strengthening of the quadriceps and hamstrings, and gait training, including stair climbing.

In this retrospective cohort study using medical records, we aimed to investigate factors influencing THC in patients undergoing primary TKA. The following variables were examined for their association with THC: age, body mass index (BMI), operative time, and Hospital for Special Surgery (HSS) knee score [14] as the knee clinical score, all of which were included as continuous variables; sex and ASA physical status [12] were included as categorical variables. The relationship between each variable and THC was analyzed using statistical methods. Additionally, multivariate analysis was performed to consider multiple variables simultaneously.

Assessment items

The following four items were assessed (all of which have detailed price settings determined by the government): in-hospital management fees which includes management fees and consumables stipulated in the insurance fee schedule (mainly hospitalization fees, medical management fees, drug costs, imaging-related costs, and meal costs), in addition to out-of-pocket expenses (mainly private room charges), excluding those during surgery; surgical fees which includes artificial joint insertion fees and autologous bone graft fees, which are determined by the medical fee schedule when deemed necessary during surgery; surgery-related expenses that includes anesthesia fees, insertion component fees, and costs for chargeable material and drugs used during surgery, such as bone cement, these costs were used to evaluate necessary expenses other than surgical fees on the day of surgery; and in-hospital rehabilitation fees which includes the total amount of insurance points per hour set by the government, accumulated from before surgery to discharge, this amount was used to evaluate rehabilitation expenses essential for functional recovery before and after surgery.

Discharge criteria

LOS until discharge to home was determined in discussions with the surgeon, rehabilitation staff, the patient, and their family. The patient's satisfaction and confidence in daily life after TKA were given the highest priority. In this study, there were no discharges owing to medical reasons on the healthcare provider's side. Following the clinical pathway provided to the patient and family before TKA, the patient was eligible for discharge from the hospital when the physical therapist confirmed their independence in stair-climbing with crutches.

Statistical analysis

Because some variables did not have a normal distribution, as determined using the Kolmogorov-Smirnov and Shapiro-Wilk tests or a Q-Q plot, these variables were analyzed using a nonparametric method. For comparisons of continuous variables, we used the Mann-Whitney U test for independent samples. Spearman’s rank correlation coefficient was used to assess relationships between two variables. The strength of the correlations was categorized as follows: strong (0.70-1.0), moderate (0.40-0.69), and weak (0.20-0.39). Factors associated with total admission fees were examined in multiple regression analysis. The candidate independent variables were included in multiple regression analysis, and significant variables were selected using a stepwise selection method. Post hoc power analyses were carried out under these circumstances. Statistical analyses were performed using R version 4.4.1 (R Foundation for Statistical Computing, Vienna, Austria) and G*power version 3.1. Values are presented as median (interquartile range, IQR), and a p-value of < 0.05 was considered statistically significant.

## Results

Post hoc power analysis revealed that the power of the Mann-Whitney U test was 67.6%, the power of Spearman's rank correlation coefficient was 87.6%, and the power of multiple regression analysis was 93.7%. All analyses were performed at a significance level of 0.05. The following results were obtained for LOS, THC, and its components:

Length of stay and total hospital cost

The median LOS was 31 days (IQR 28; 38) (Table 1), and the median THC was JPY 2,268,670 (IQR 2160233; 2463608) or approximately USD 15,125 (IQR 14,402; 16,424), based on an exchange rate of USD 1=JPY 150.

**Table 1:** Patients’ background information BH: body height; BW: body weight; BMI: body mass index; HSS: Hospital for Special Surgery [14] Values are expressed as n or median (25th percentile, 75th percentile).

Variables	Median (interquartile range)	Range
Age (year)	75 (71, 78)	58 to 88
BH (cm)	151 (146, 155)	137 to 172
BW (kg)	60 (54, 68)	44 to 105
BMI (kg/m²)	26 (24, 29)	17 to 42
Operation time (min.)	55 (50, 62)	39 to116
HSS score (points)	47 (39, 54)	10 to 70
Length of hospital stay (days)	31 (28, 38)	17 to 63

Breakdown of total hospital cost

The breakdown of the THC was as follows (total amount and percentage relative to THC): in-hospital management costs JPY 582,970 (IQR 555,995; 614,112) or 25.7% (582,970/2,268,670) (IQR 24.5%; 27.1%); surgical fees JPY 545,194 (IQR 502,067; 572,569) or 24.0% (545,194/2,268,670) (IQR 22.1%; 25.2%); surgery-related expenses JPY 580,619 (IQR 528,135; 609,834) or 25.6% (580,619/2,268,670) (IQR 23.3%; 26.9%); and in-hospital rehabilitation fees JPY 560,361 (IQR 512,753; 616,183) or 24.7% (560,361/2,268,670) (IQR 22.6%; 27.2%), with each item accounting for a nearly equal proportion.

Factors affecting total hospital cost

Patient Factors

A Mann-Whitney U test was performed to compare the distributions of values by sex (men: 2,335,280 (IQR 2,242,005; 2,609,815); women: 2,264,261 (IQR 2,154,840; 2,413,980)) and between ASA grades (I: 2,229,980 (IQR 2,174,520; 2,335,280); II: 2,315,620 (IQR 2,158,440; 2,488,470)). No statistically significant difference was observed in either comparison. Age showed a weak positive correlation with THC (r=0.364, p< 0.001) (Table 2); however, sex (p=0.144), BMI (p=0.090), ASA grade (p=0.150), operative time (p=0.611), and HSS score (p=0.264) did not affect THC (Table 2).

**Table 2:** Univariate analysis (I) Correlation between total hospital charges (median: JPY, 2,268,670) and patient factors and length of stay as a medical factor using Spearman's rank correlation r: correlation coefficient; BMI: body mass index; ASA: American Society of Anesthesiologists [12]; HSS: Hospital for Special Surgery [14]

Variables	Median	p-value	r
Age	75 year	< 0.001	0.364
SEX (Men)	JPY 2,335,280	0.144	0.147
SEX (Women)	JPY 2,264,261		
BMI	26 kg/m²	0.090	-0.017
ASA (I)	JPY 2,229,980	0.150	0.145
ASA (II)	JPY 2,315,620		
Operation time	55 min	0.611	-0.051
HSS score	47 points	0.264	-0.112
Length of hospital stay	31 days	< 0.001	0.797

Healthcare Provider Factors

LOS showed a strong positive correlation with THC (r=0.797, p<0.001) (Table 2). Regarding relationships with the four components of THC, reflecting the fee-for-service system, in-hospital management costs (r=0.720, p< 0.001) and rehabilitation fees (r=0.751, p< 0.001) showed a positive correlation with prolonged hospitalization (Table 3). However, surgical fees (r=−1.000, p< 0.001) and surgery-related expenses (r=−0.972, p< 0.001), which are fixed, showed a negative correlation; their proportions were relatively decreased with longer LOS (Table 3).

**Table 3:** Univariate analysis (II) Correlation between total hospital charges (median value, JPY 2,268,670) and breakdown of its four components using Spearman's rank correlation r: correlation coefficient

Each component	Median charges	p-value	r
In-hospital management fees	JPY 582,970	< 0.001	0.720
Surgical procedure fees	JPY 545,194	< 0.001	-1.000
Surgery-related expenses	JPY 580,619	< 0.001	-0.972
In-hospital rehabilitation fees	JPY 560,361	< 0.001	0.751

Multiple regression analysis with stepwise selection

Multiple regression analysis revealed significant correlations between THC and several variables. LOS (β=0.31, p<0.001), along with the associated inpatient management fees (β=0.66, p<0.001) and rehabilitation fees (β=0.10, p< 0.001), demonstrated a positive correlation with THC (Table 4).

**Table 4:** Results of multiple regression analysis with stepwise selection SE: standard error; sig: significance; CI: confidence interval

Variables	Median value	B	SE	β	sig.	95% CI
(Intercept)		103100.00	4484.00	0.00	<0.001	95685.62- 110581.00
In-hospital management fees	JPY 582,970	1.37	0.09	0.66	<0.001	1.22- 1.51
In-hospital rehabilitation fees	JPY 560,361	17.94	5.02	0.10	<0.001	9.60- 26.28
Length of hospital stay	31 days	902.60	122.70	0.31	<0.001	698.79- 1106.40

## Discussion

Two key findings emerged from this study. First, we demonstrated that the total cost of TKA hospitalization is significantly influenced by LOS, a healthcare provider-related factor, as well as patient age. Second, regarding the cost structure, the four cost components were nearly equally distributed. Furthermore, with increasing LOS, the proportions of in-hospital management and rehabilitation fees (which are reimbursed on a fee-for-service basis) were increased. Conversely, the proportions of surgical fees and surgery-related expenses, which are fixed, were relatively decreased.

This study revealed that THC for TKA is significantly influenced by LOS. This is likely because Japan's fee-for-service reimbursement system ties cost to the services provided; as LOS increases, costs corresponding to services such as in-hospital management and rehabilitation also increase. However, fixed costs such as surgical fees and surgery-related expenses (e.g., implants, instruments, anesthesia) were proportionally decreased. This revealed an important factor explaining the difference in hospitalization trends among Western countries, which have a bundled payment system and a tendency toward shorter hospital stays (from outpatient [15,16] two to three nights [17-19]), and Japan, which has a fee-for-service system and a tendency toward longer hospital stays (five to nine weeks [9,10,11,20,21]). In Western countries with bundled payments, medical institutions have a financial incentive to shorten LOS so as to maximize profits. Conversely, in Japan, with a fee-for-service system, medical institutions may have a financial incentive to lengthen LOS to maximize revenue per patient. Whereas the fee-for-service system may create incentives for longer hospital stays, some Japanese institutions are actively pursuing strategies to optimize LOS without compromising patient care [9,20].

Analysis of the breakdown of in-hospital TKA costs revealed that the four components, in-hospital management fees, surgical fees, surgery-related expenses, and in-hospital rehabilitation fees, each accounted for nearly equal proportions. From a patient-centered perspective, this suggests that TKA treatment not only requires surgery but also comprehensive care, including appropriate pre- and postoperative in-hospital management and rehabilitation, to optimize patient outcomes such as pain relief, functional recovery, and overall satisfaction. However, TKA treatment in Western countries with healthcare systems based on global budgets or fixed payments per case has recently been focused on shortening hospital stays [15-17]. Additionally, some studies have reported that direct guidance by therapists in postoperative TKA rehabilitation has limited benefits in terms of both clinical outcomes and cost-effectiveness, leading to a focus on more efficient care pathways [22,23]. It should be noted that although the analysis of in-hospital TKA cost components highlights the importance of perioperative management, and whereas shorter stays are desirable, the findings also suggest that shortening hospital stays requires a comprehensive evaluation from the patient's perspective, including treatment effectiveness such as mobility and pain levels at discharge, as well as patient satisfaction. Ultimately, optimizing TKA costs requires a balanced approach that considers both efficiency and the comprehensive needs of the patient to ensure positive outcomes and value.

Regarding patient factors, age demonstrated a weak positive correlation with THC, suggesting a trend toward increased costs with advancing age. This likely stems from the observed increase in LOS among older patients [9,24,25], which subsequently drives up THC. Although Enhanced Recovery After Surgery protocols [26], which use evidence-based medicine, are widely implemented [27,28], this study underscores the need for even more efficient in-hospital management and postoperative rehabilitation systems to control THC by optimizing LOS, particularly in geriatric patients. Improved discharge planning and support may further contribute to shorter LOS and reduced costs [29]. However, THC is influenced by a multitude of factors beyond patient characteristics, including the institution's care pathways and the healthcare reimbursement model. Therefore, future research applying a multifaceted approach is crucial to enhance the quality and value of TKA.

This study has several limitations. First, its retrospective, single-institution design limits how broadly we can apply these findings to other hospitals and patient populations. Multicenter, prospective studies with larger and more diverse patient groups are needed to confirm these results. Second, we did not specifically account for variations in resource allocation between hospitals. This includes differences in staffing levels (e.g., number of nurses and rehabilitation staff) and available bed capacity. Furthermore, we did not evaluate the influence of different reimbursement systems (such as the DPC/PDPS and fee-for-service models) on total inpatient costs. Future studies should investigate how these factors, including the choice of reimbursement system, impact TKA costs. Third, although we examined the main cost components, we did not fully investigate the specific expenses within each category, nor did we assess how efficiently resources were used. These detailed analyses are essential for pinpointing areas where costs can be reduced and efficiency improved. Despite these limitations, this study is valuable as it is one of the few to thoroughly analyze total in-hospital expenses for TKA surgery in Japan, particularly within the context of its fee-for-service system.

## Conclusions

In this study, we elucidated the cost structure of inpatient care following TKA in Japan. We revealed that prolonged LOS, reflective of the fee-for-service system, and the associated increase in hospital management and rehabilitation fees have a significant impact on THC. Conversely, under patient-driven discharge decisions, we observed a relatively even distribution of cost components, suggesting the need for comprehensive inpatient care over a certain period that includes pain control and functional recovery rehabilitation. Future research should focus on identifying and implementing best practices for TKA care that balance cost-effectiveness with optimal patient outcomes.
